# Biomechanical analysis of four different meniscus suturing techniques for posterior meniscal root pull‐out repair: A human cadaveric study

**DOI:** 10.1002/jeo2.70020

**Published:** 2024-09-23

**Authors:** Ting‐Yu Chang, Tai‐Hua Yang, Kuan‐Yu Lin

**Affiliations:** ^1^ Department of Orthopedic Surgery, National Cheng Kung University Hospital, College of Medicine National Cheng Kung University Tainan Taiwan; ^2^ Department of Biomedical Engineering, College of Engineering National Cheng Kung University Tainan Taiwan; ^3^ Department of Orthopedic Surgery Kaohsiung Veterans General Hospital Kaohsiung City Taiwan; ^4^ Department of Nursing Meiho University Pingtong Taiwan

**Keywords:** knee, meniscus root, pull‐out repair, root tear, slip‐knot

## Abstract

**Purpose:**

To compare the biomechanical properties of the slip‐knot technique with three other transtibial pullout suture repair constructs for meniscal root tears.

**Method:**

Thirty‐two fresh‐frozen cadaveric menisci were randomly allocated to four meniscus‐suture fixation constructs: Two simple‐sutures (TSS), two slip‐knot (TSK) sutures, two cinch‐loop (TCL) sutures, and two modified Mason–Allen (TMMA) sutures. Cyclic loading from 5 to 20 N was conducted for 1000 cycles at 0.5 Hz, and then loaded to failure at 0.5 mm/s. Parametric data (displacement during cyclic loading, ultimate load, yield load, and displacement at failure) were analysed using a one‐way analysis of variance (ANOVA), whereas nonparametric data (stiffness) were analysed using the Kruskal–Wallis test.

**Results:**

After 1000 cycles, the TCL construct significantly displaced the most (mean ± SD, 6.78 ± 1.32 mm; *p* < 0.001), followed by the TMMA (2.83 ± 0.90 mm), TSK (2.33 ± 0.57 mm), and TSS (2.03 ± 0.62 mm) groups. On ultimate failure load, there was no significant difference between the TSK group (123.48 ± 27.24 N, *p* > 0.05) and the other three groups (TSS, 94.65 ± 25.33 N; TMMA, 168.38 ± 23.24 N; TCL, 170.54 ± 57.32 N); however, it exhibited the least displacement (5.53 ± 1.25 mm) which was significantly shorter than those of the TCL (11.82 ± 4.25 mm, *p* < 0.001) and TMMA (9.53 ± 2.18 mm, *p* = 0.03) constructs. No significant difference in stiffness was observed among the four meniscus‐suture constructs.

**Conclusion:**

The slip‐knot technique has proven to be a simple, yet robust and stable meniscal root fixation option; moreover, it exhibited superiority over the more complex modified Mason–Allen suture construct in resisting displacement at the ultimate failure load.

**Level of Evidence:**

Not applicable.

**Abbreviations**
TCL: two cinch‐loop
TMMA: two modified Mason–Allen
TSK: two slip‐knot
TSS: two simple‐suture

## INTRODUCTION

The effect of a posterior root tear on knee joint biomechanics is equivalent to that of a total meniscectomy, resulting in a substantial increase in peak contact pressure within the tibiofemoral compartment [12, 16, 17, 36]. Meniscal root repair is essential to restore meniscal function, enhance joint stability, improve functional outcomes, and prevent joint degeneration [12, 13, 18, 21, 33, 36]. Biomechanical studies have indicated that a transtibial pullout suture repair construct can restore tibiofemoral contact mechanics to those of the intact state at time zero [7, 20, 29, 31]. Additionally, clinical studies have demonstrated that the transtibial suture pullout technique effectively reduces meniscus extrusion, a predictive factor for unsatisfactory clinical outcomes and early‐onset osteoarthritis of the knee joint [14, 26]. However, the interface between the meniscus and the suture configuration is the primary site of failure due to suture cutout from the meniscus. Hence, optimizing the stability of this interface is a key focus of research [4, 28].

The biomechanical strength of various meniscal suture fixation techniques has been examined in several studies, indicating a correlation between the complexity of repair constructs and increased ultimate failure loads [1, 21, 23, 25]. The two simple‐suture (TSS) pattern is preferred because it provides substantial resistance to cyclic displacement, although it has lower yield and ultimate failure loads than other methods [1, 17]. By contrast, the modified Mason–Allen configuration offers a superior ultimate failure load [2, 8, 21]. However, modified Mason–Allen involves multiple piercings of the meniscus, posing a risk of damage to the meniscus and articular cartilage, and suture management following the use of this technique is arthroscopically challenging and time consuming. Krych et al. [19] demonstrated a simple technique using a cinch‐loop suture for pullout repair, providing a comparable ultimate failure load to that of a locking loop with less cyclic displacement. Nevertheless, these commercially manufactured closed‐loop‐ended sutures are costly and may not be readily available to all surgeons.

Chen et al. [5] introduced a slip‐knot technique that offers an efficient construct that combines the simplicity of the cinch‐loop technique with the stability of the locking loop technique for posterior meniscal root tears. However, the biomechanical strength of this technique has not been verified in vitro. The present study compared the biomechanical properties of the slip‐knot technique with those of the TSS, modified Mason–Allen, and cinch‐loop suture constructs for meniscal root tears. The authors hypothesized that the slip‐knot technique is a biomechanically compatible option for meniscal root pullout repair.

## MATERIALS AND METHODS

### Specimen acquisition

The authors harvested 32 freshly frozen medial and lateral posterior meniscal roots from 16 human cadaveric knee specimens (eight male and eight female) with an average age of 76 ± 7 years (range: 62–87 years). On the basis of other biomechanical studies [1, 9, 15, 24, 37], a sample size of eight meniscal roots per group was determined to be adequate to achieve the desired power. Except for the TSS group, which had five medial and three lateral menisci, all groups had an even distribution of four medial and four lateral menisci to minimize the influence of the medial–lateral discrepancy. A post hoc power analysis confirmed the adequacy of this sample size.

### Specimen preparation

Both medial and lateral menisci were resected entirely from each knee specimen, but only the posterior roots were sutured and tested in this study. All menisci were frozen until 24 h before biomechanical testing, thawed at room temperature, and maintained in saline‐soaked gauze to prevent desiccation. Through a computer software (Microsoft Excel)‐generated simple randomization system, 32 menisci were randomly assigned at a 1:1:1:1 ratio to one of four testing groups (*n* = 8 per group).

### Suture material

All suture configurations used two nonabsorbable #2 FiberWire (Arthrex; Naples, Florida, USA) sutures except for the cinch‐loop technique, which used two nonabsorbable #2 FiberLink (Arthrex; Naples, Florida, USA) sutures. The tensile strengths of both suture materials were investigated to minimize selection bias.

### Suture techniques

**Figure 1:**
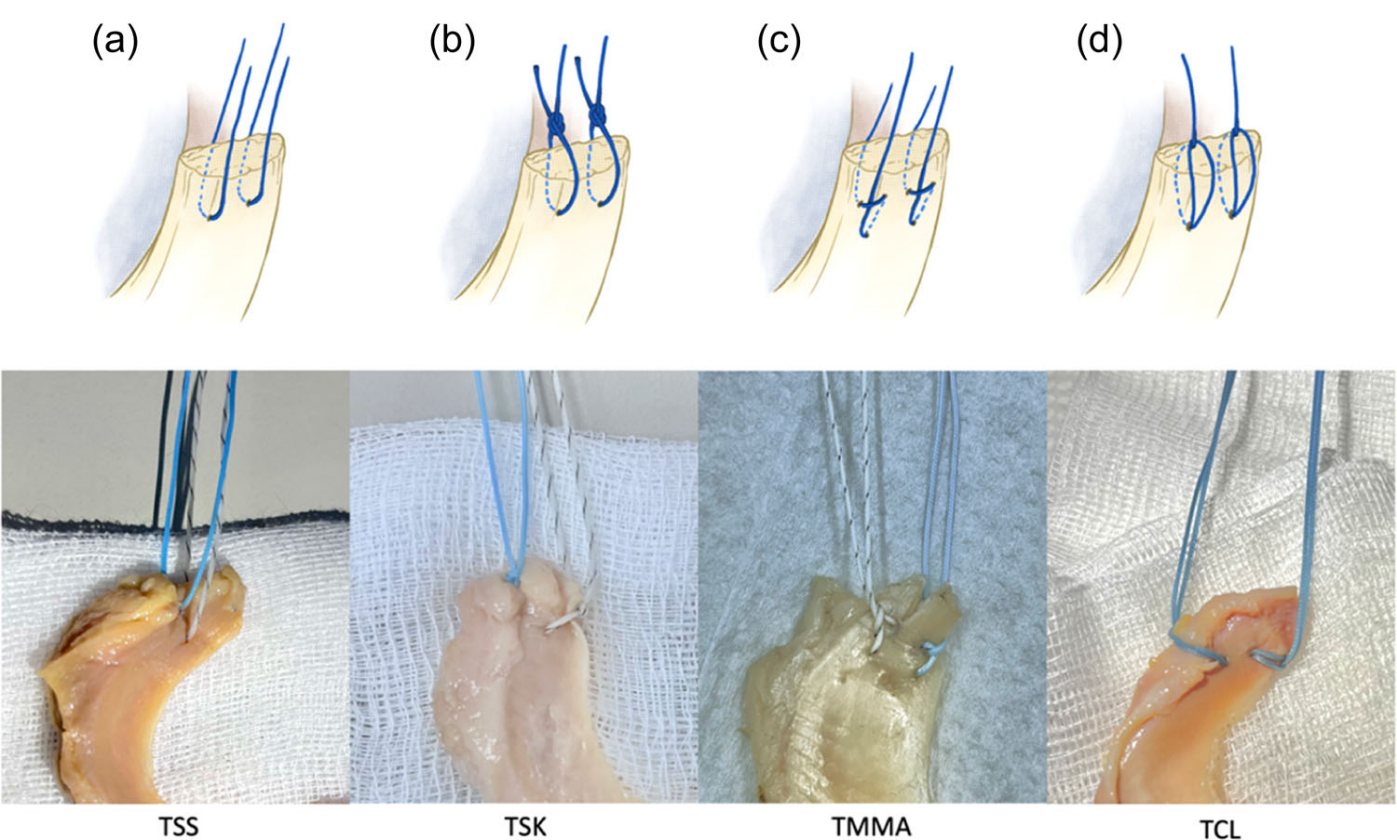
Schematic illustrations and corresponding images of the four meniscus‐suture configurations in this study. (a) Two simple‐suture (TSS) configuration, (b) two slip‐knot (TSK) configuration, (c) two modified Mason–Allen (TMMA) configuration, and (d) two Cinch‐Loop (TCL) configuration.

The present study evaluated four suturing techniques (Figure 1). The efficacy of meniscal repair constructs may vary depending on the suture configuration and material and the number and position of sutures used [30]; therefore, to ensure standardization, all suturing procedures were performed by a single senior‐level, board‐certified orthopaedic surgeon (K.Y.L.) who had undergone fellowship training for sports medicine. Every configuration consisted of two sutures placed 5 mm apart anteroposteriorly, 7 mm from the root edge, using a curved tapered needle.

The TSS configuration followed the protocol developed by Feucht et al. [8] (Figure 1a), which is considered the clinical standard for pullout suture repair due to its technical simplicity and biomechanical resistance to displacement [8, 9, 10, 21].

**Figure 2:**
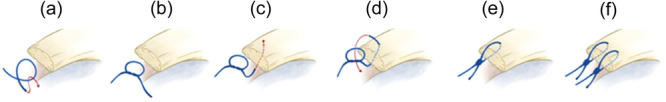
A detailed step‐by‐step illustration of the slip‐knot technique. (a, b) Creating a half‐hitch loop at the middle of the suture first; (c) the working limb pierces through the meniscus from underneath and passes through the half‐hitch loop to form a slipped knot (d). (e) By pulling on the working limb, the knot then slides and tightens onto the meniscus. (f) Final construct.

The two slip‐knot (TSK) configuration replicated the technique developed by Chen et al. [5] (Figure 1b). This procedure initially involves creating a half‐hitch loop in the middle of the suture. One end of the suture (the working limb) is then passed through the half‐hitch loop to form a slipped knot after piercing the meniscus using a curved tapered needle. Finally, the working limb is pulled to slide the knot onto the meniscus and tighten it (Figure 2).

The two modified Mason–Allen (TMMA) configuration (Figure 1c) followed techniques described by Feucht et al. [8] and Lee et al. [22]. The initial step involves creating a horizontal mattress suture by passing the suture twice through the meniscus, resulting in a horizontal suture loop on the superior meniscal surface. Subsequently, one suture limb is medially threaded through the meniscus deep and in the centre of the horizontal suture loop.

The TCL technique was modelled after that developed by Krych et al. [19] using two #2 FiberLink (Arthrex) sutures (Figure 1d). After piercing the meniscal root from below, the free end of the suture passes through the loop end, which then slides onto the meniscal root to form a cinch‐loop configuration.

### Mounting

**Figure 3:**
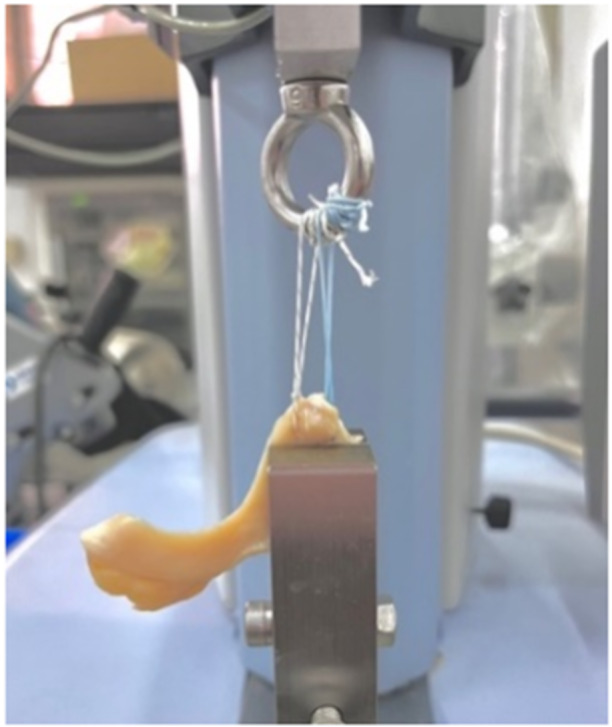
The biomechanical testing set‐up.

The mounting protocol used in this study was adapted from other published methods [4, 8, 10]. In this protocol, a tissue clamp secured the meniscal body 10 mm away from the suture insertion point in the meniscus. The clamp was attached to the base of the mechanical testing machine (EZ‐SX; Shimadzu), and the end of the suture was fastened around a hook linked to the machine's linear actuator (Figure 3). Each suture's two limbs were wrapped four times each around the O‐hook and tightly fastened with multiple knots. This wrapping method was pre‐tested and proved to have strength superior to that of the suture material itself, which has a failure load of approximately 200 N. Before each test, the suture length between the hook and the meniscus insertion point was adjusted to 40 mm. The mounting and testing procedures were conducted under standard room temperature conditions, and the specimens were maintained in a constant moistened state with saline solution spray.

### Biomechanical testing

Each suture configuration underwent an identical cyclic tensioning protocol. The cyclic loading procedure involved applying a preload of 2 N for 10 s, followed by cyclic loading between 5 and 20 N for 1000 cycles at a frequency of 0.5 Hz. Displacements were monitored and recorded at the actuator of the tensile testing machine at cycles 1, 100, 500, and 1000. The measurement error of the testing machine was certified by Shimadzu to be either 0.1% of the indicated value or 0.01 mm, whichever was larger. More detailed information can be obtained from the official online operation manual (https://www.shimadzu.com/an/products/materials-testing/uni-ttm/ez-test/index.html). The observed displacement after cyclic loading indicates the change in the total length of the construct, representing the distance between the clamp and the hook. This testing protocol was adapted from other biomechanical evaluations of root repair and simulated postoperative loads in vivo after repair [4, 21].

**Figure 4:**
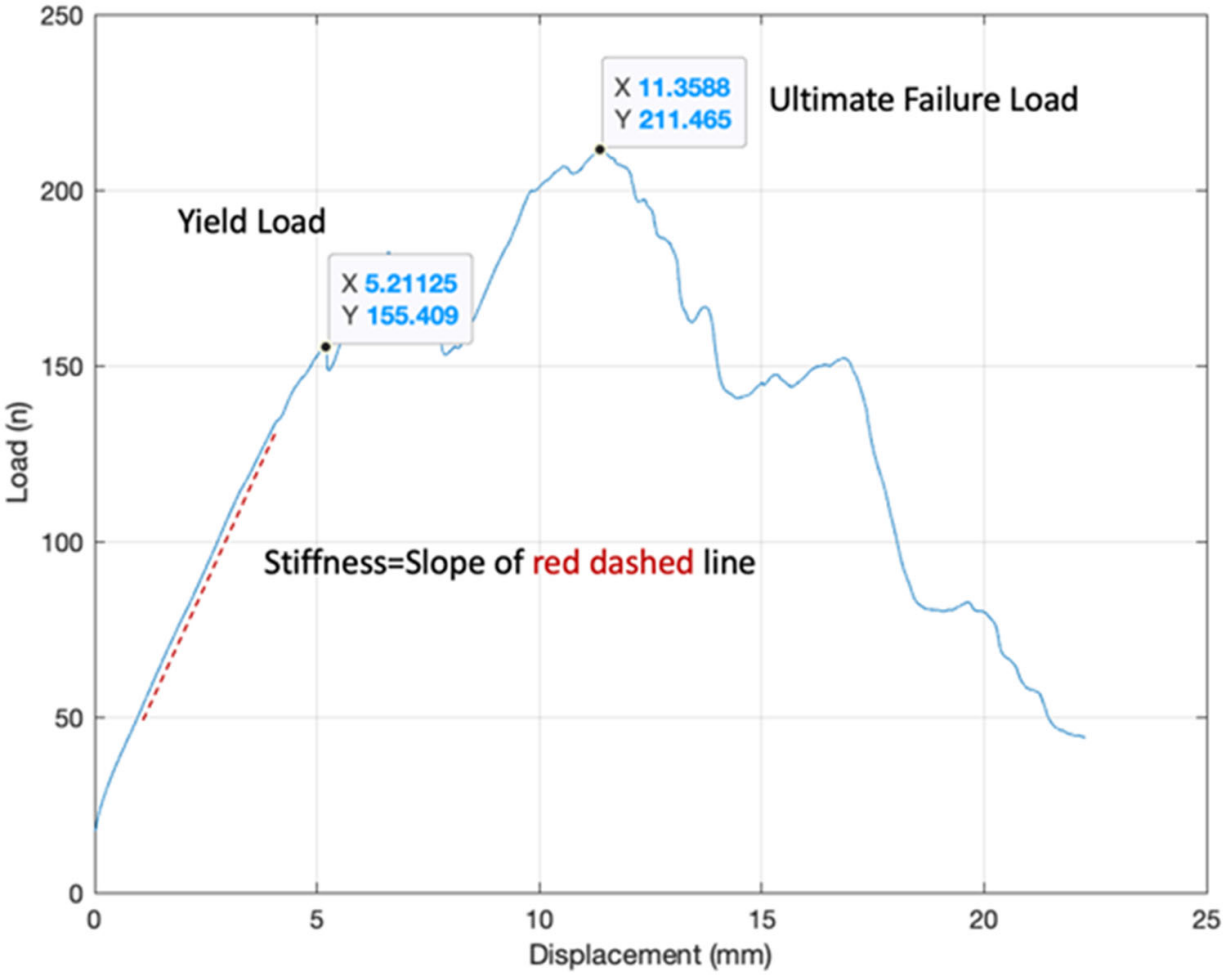
Representative of the load–displacement curve. The stiffness was calculated as the slope of the red dashed line.

Following the cyclic loading test, a load‐to‐failure test was conducted at a rate of 0.5 mm/s until failure. Indices measured for each construct included yield load, displacement at the yield load, ultimate failure load, displacement at ultimate failure, and construct stiffness. Stiffness is a measure of the ability of a construct to resist deformation when a force is applied, which were defined similarly to those in previous study by Vertullo et al. [34]. Stiffness values were determined by the slope of the load‐displacement curve during load to failure test as shown in Figure 4.

### Statistical analysis

A post hoc power analysis was performed using G*Power 3.1 software (Franz Paul) to determine the power of the current study. Based on the results of yield load, ultimate failure load, stiffness, and displacement after 1000 cycles, effect sizes of 0.873, 0.883, 0.225, and 2.14 were calculated, respectively. With these effect sizes and an *α* of 0.05, the calculated power was 0.98, 0.98, 0.15, and 1.00, respectively, for eight samples per group.

The normality of the distribution of all test variables was assessed using the Kolmogorov–Smirnov test. Parametric data (displacement during cyclic loading, ultimate load, yield load, and displacement at failure) were analysed using a one‐way analysis of variance (ANOVA) and post hoc Tukey's honest significant difference (HSD) test, while nonparametric data (stiffness) were analysed using Kruskal–Wallis test followed by the Mann–Whitney *U* test. The level of significance was set at *p* < 0.05. All statistical analyses were performed using SPSS software version 17.0 (IBM‐SPSS).

## RESULT

### Suture material strength

The ultimate loads of the two suture materials employed in this study (#2 FiberWire™ Suture and #2 FiberLink™ Suture, Arthrex Inc.) were evaluated to confirm that any differences observed were not confounded by the strength differences of the suture materials themselves. Six sutures of each were tested, and the results showed that there was no statistically significant difference in the mean ultimate failure load between the two (213.9 ± 38.87 N and 197.8 ± 34.47 N for #2 FiberWire™ and #2 FiberLink™, respectively; *p* = 0.42).

### Cyclic displacement

**Figure 5:**
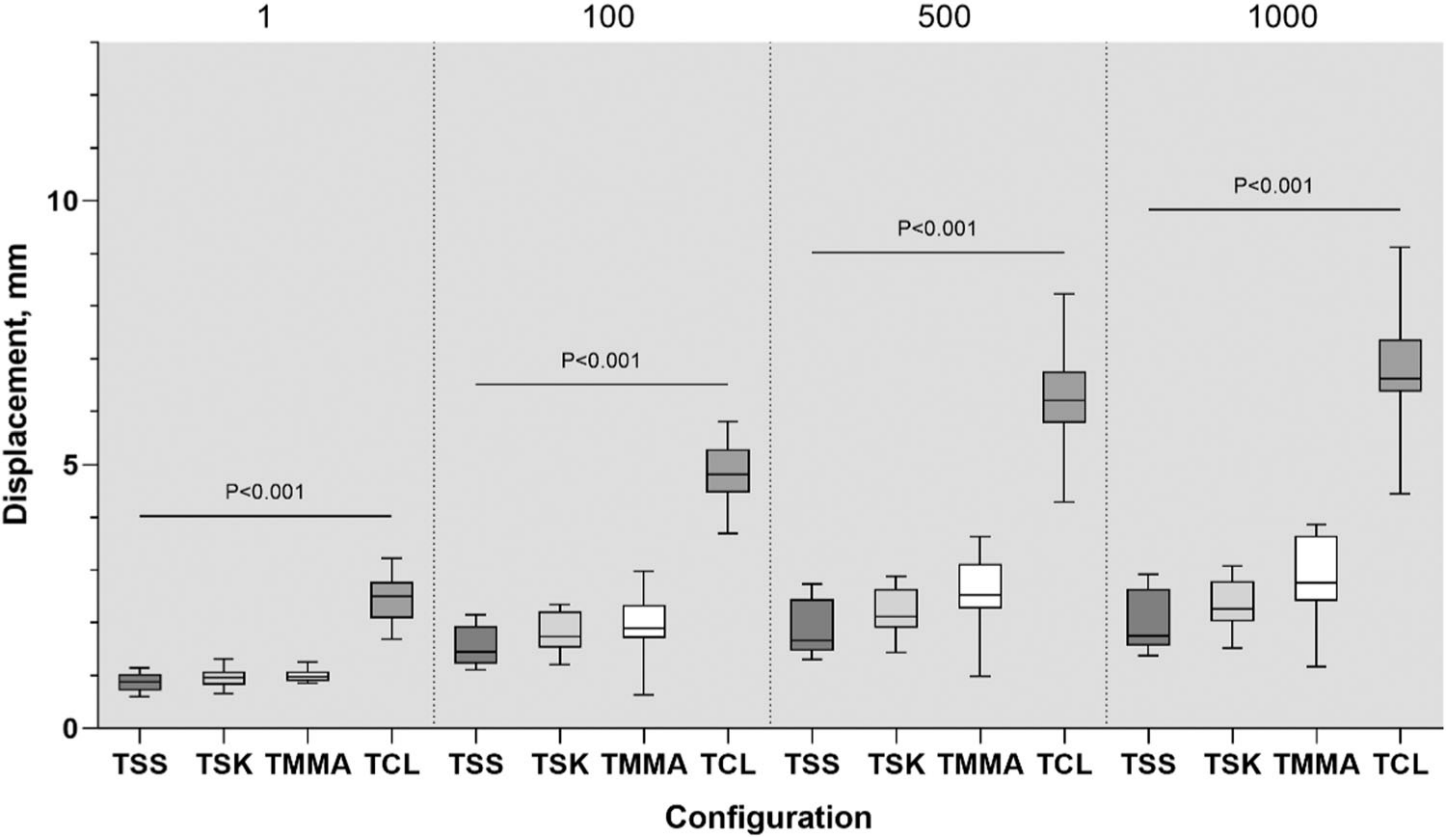
This box plot depicts the displacement observed during cyclic loading at 100, 500, and 1000 cycles. Overhead bars represent statistically significant interconstruct comparison (*p* < 0.05). TCL, two cinch‐loop; TMMA, two modified Mason‐Allen; TSK, two slip‐knot; TSS, two simple‐suture.

No failures occurred during the cyclic loading tests for any of the four constructs. The displacements observed for each testing group after preconditioning for 1, 100, 500, and 1000 cycles are depicted in Table 1 and Figure 5. Post hoc comparisons indicated that only TCL technique produced statistically significant greater displacement at the end of cycles 1, 100, 500, and 1000 (*p* < 0.001 in all cyclic loading tests), whereas no significant difference was observed among the TMMA, TSK, and TSS techniques at the conclusion of each cyclic loading test.

**Table 1:** Displacement during cyclic loading.

	Displacement (mm)			
Group	After 1 cycle	After 100 cycles	After 500 cycles	After 1000 cycles
TSS	0.88 ± 0.20 (0.71–1.04)	1.57 ± 0.40 (1.23–1.90)	1.90 ± 0.56 (1.43–2.37)	2.03 ± 0.62 (1.51–2.54)
TSK	0.97 ± 0.23 (0.78–1.16) [10.5%]	1.79 ± 0.44 (1.42–2.16) [14.4%]	2.19 ± 0.53 (1.75–2.63) [15.2%]	2.33 ± 0.57 (1.86–2.81) [15.1%]
TMMA	1.01 ± 0.14 (0.89–1.13) [15.2%]	1.95 ± 0.68 (1.38–2.51) [24.5%]	2.58 ± 0.82 (1.89–3.27) [35.5%]	2.83 ± 0.90 (2.08–3.59) [39.7%]
TCL	2.45 ± 0.51 (2.02–2.88) [180%]^a^	4.83 ± 0.72 (4.22–5.42) [208%]^b^	6.26 ± 1.13 (5.32–7.20) [229%]^c^	6.78 ± 1.32 (5.68–7.89) [234%]^d^

*Note*: Data are shown as mean ± standard deviation (95% confidence interval). Values in square bracket are the percentages of greater displacement compared to the TSS technique.

Abbreviations: TCL, two cinch‐loop; TMMA, two modified Mason–Allen; TSK, two slip‐knot; TSS, two simple‐suture.

^a^ Significant difference compared with TSS after 1 cycle (*p* < 0.001).

^b^ Significant difference compared with TSS after 100 cycles (*p* < 0.001).

^c^ Significant difference compared with TSS after 500 cycles (*p* < 0.001).

^d^ Significant difference compared with TSS after 1000 cycles (*p* < 0.001).

### Load to failure

**Figure 6:**
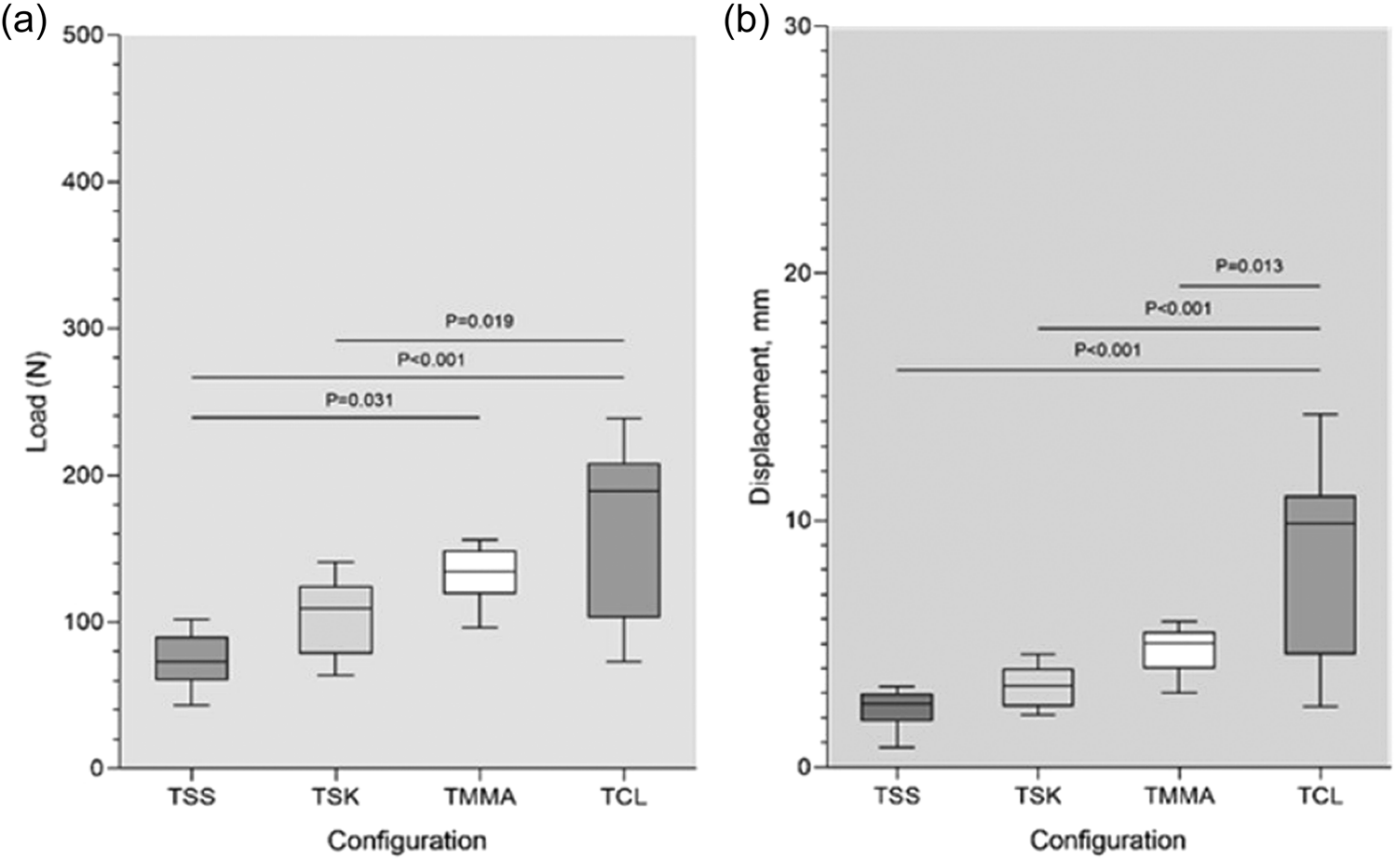
This box plot displays the yield load (a) and displacement at yield load (b). Overhead bars represent statistically significant interconstruct comparison (*p* < 0.05). TCL, two cinch‐loop; TMMA, two modified Mason–Allen; TSK, two slip‐knot; TSS, two simple‐suture.

Comparisons of the yield load and the ultimate failure load are shown in Table 2. Compared to the standard TSS configuration, the TMMA and TCL had significantly greater mean yield loads, with increases of 60.26 N (or 81.8%) and 90.4 N (or 122%), respectively. Post hoc comparisons revealed that the TCL construct had a significantly greater yield load than did the TSS and TSK constructs (*p* < 0.001 and *p* = 0.019, respectively); however, the TCL construct also exhibited significantly greater displacement at yield load than did the TMMA, TSK, and TSS constructs (*p* = 0.013, *p* < 0.001, and *p* < 0.001, respectively), while no significant differences were detected among the latter three (Figure 6).

**Table 2:** Yield load, displacement at yield load, ultimate failure load, displacement at failure and stiffness.

	TSS	TSK	TMMA	TCL
Yield load (N)	73.64 ± 22.12 (55.1–92.1)	102.90 ± 28.42 (79.1126.7)[39.7%]	133.90 ± 21.08 (116.3–151.5)[81.8%]^a^	164.04 ± 65.05 (109.7–218.4)[122%]^b^, ^c^
Displacement at yield load (mm)	2.30 ± 0.94 (1.51–3.08)	3.30 ± 0.92 (2.52–4.07)[43.7%]	4.74 ± 0.83 (4.04–5.42)[106%]	8.57 ± 4.26 (5.00–12.13)[273%]^d^
Ultimate failure load (N)	94.65 ± 25.33 (73.5–115.8)	123.48 ± 27.24 (100.7–146.2)[30.5%]	168.38 ± 23.24 (148.9–187.8)[77.9%]^e^	170.54 ± 57.32 (122.6–218.4)[80.2%]^f^
Displacement at ultimate failure (mm)	5.67 ± 2.19 (3.84–7.50)	5.53 ± 1.25 (4.49–6.58)[−2.4%]	9.53 ± 2.18 (7.71–11.36)[68.1%]^g^	11.82 ± 4.25 (8.27–15.36)[108%]^h^
Stiffness (N/mm)	23.84 ± 10.65 (17.40–30.29)	24.95 ± 4.01 (18.51–31.39)[4.64%]	23.15 ± 2.98 (16.71–29.59)[−2.9%]	19.61 ± 13.33 (13.17–26.05)[−18%]

*Note*: Data are shown as mean ± standard deviation (95% confidence interval). Values in square bracket are the percentages of greater displacement or loading force compared to the TSS technique. Abbreviations: TCL, two cinch‐loop; TMMA, two modified Mason–Allen; TSK, two slip‐knot; TSS, two simple‐suture.

^a^ TMMA had significantly greater yield load compared to TSS (*p* = 0.021).

^b^ TCL had significantly greater yield load compared to TSS (*p* < 0.001).

^c^ TCL had significantly greater yield load compared to TSK (*p* = 0.018).

^d^ TCL had a significantly greater displacement at yield load compared to TSS, TSK, and TMMA (*p* < 0.001, *p* < 0.001, and *p* = 0.011).

^e^ TMMA had significantly greater ultimate failure load compared to TSS (*p* = 0.002).

^f^ TCL had significantly greater ultimate failure load compared to TSS (*p* = 0.001).

^g^ TMMA had significantly greater displacement at ultimate failure load compared to TSS and TSK (*p* = 0.037 and *p* = 0.03).

^h^ TCL had significantly greater displacement at ultimate failure load compared to TSS and TSK (*p* = 0.001 and *p* < 0.001).

**Figure 7:**
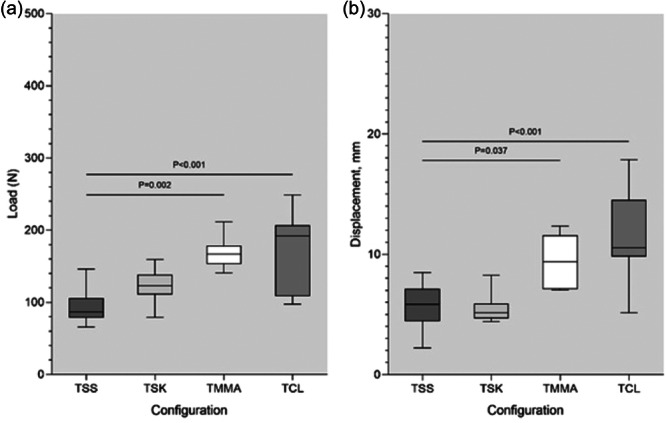
This box plot displays the ultimate failure load (a) and displacement at ultimate failure load (b). Overhead bars represent statistically significant interconstruct comparison (*p* < 0.05). TCL, two cinch‐loop; TMMA, two modified Mason–Allen; TSK, two slip‐knot; TSS, two simple‐suture.

The ultimate failure loads of both the TCL (170.54 ± 57.32 N) and TMMA (168.38 ± 23.24 N) constructs were significantly greater than that of the TSS configuration (94.65 ± 25.33 N) (*p* < 0.001 and *p* = 0.002, respectively) but not significantly greater than that of the TSK configuration (*p* = 0.065 and *p* = 0.084, respectively) (Figure 7). Comparing the displacements at their respective ultimate failure loads, the TSK configuration exhibited the least elongation (5.53 ± 1.25 mm), which was significantly less than those of the TCL (11.82 ± 4.25 mm, *p* < 0.001) and TMMA (9.53 ± 2.18 mm, *p* = 0.03) constructs. Suture cut‐out was the cause of failure in all constructs in this study, which reverberated the results of previous studies [4, 21].

### Stiffness

The result showed there was no statistically significant difference among the four fixation methods (Table 2).

## DISCUSSION

This present study found that among the four pull‐out suture repairs for posterior meniscal root tears, the technically simpler TSK configuration exhibited significantly less displacement at an ultimate failure load that was comparable to that of the more complex TMMA technique. Furthermore, although the results were nonsignificant, the TSK technique yielded favourable outcomes in yield load, ultimate failure load, displacement at ultimate failure load, and stiffness compared with the standard TSS technique. By contrast, despite technically emulating the TSK technique, the TCL technique yielded the greatest displacements at the end of all cyclic loading tests.

An ideal suture repair construct should provide low displacement, high stiffness, high yield, and high ultimate failure load to maintain the reattached meniscus root in place during the healing process. Displacement at the meniscus–suture interface during the early postoperative period may result in nonanatomic (more peripheral) healing of the meniscus root, leading to diminished meniscus function [32]. Studies have demonstrated that the threshold for suture elongation required to compromise meniscal function is 3 mm [8, 21]. In the present study, the TCL and TMMA configurations both demonstrated displacements that approached or exceeded the 3 mm threshold at the end of 1000 cycles. The TSS and TSK techniques exhibited significantly less cyclic displacement than did the TCL technique which exceeded the 3 mm threshold (4.83 mm) after only 100 cycles. Bachmaier et al. suggested this increase in cyclic displacement could be attributed to the overlapping suture‐lengthening effects of cinch‐loop–based meniscus root fixation with suture cut‐through of the meniscus and continual cinch‐loop tightening during repetitive loading [2]. Additionally, the TSS construct consistently exhibited the least displacement after each cyclic loading test, likely because forces were distributed more uniformly across the meniscus–suture interface. The TSK technique was as stable as the standard TSS technique, and no significant difference in cyclic displacement was observed between the two. Furthermore, both the TCL and TMMA configurations exhibited greater displacements at their respective ultimate failure loads than did the TSK and TSS configurations, a finding consistent with those of previous studies that intricate suture constructs offer superior ultimate failure loads but typically result in greater displacements [1, 2, 8, 21, 34].

All specimens failed through the suture cutout at the suture–meniscal interface, a finding consistent with that of another study [28]. This result emphasizes that the integrity of the repaired meniscal root depends on the suture–meniscal interface. This study analysed the tensile strength of various suturing techniques at time zero in vitro rather than at the completion of the healing process in vivo. Moreover, a threshold force of only 30 N has been shown to be sufficient for early postoperative rehabilitation [11, 25], suggesting that as long as the yield load and ultimate load of a suture configuration exceed this threshold, that construct is a viable option; therefore, all four suturing techniques in the present study are feasible suturing methods for posterior root repair. However, the TCL technique exhibited a significantly greater yield load than did the TSK and TSS techniques, while no significant difference was observed between the TSK and TMMA techniques. Similarly, the ultimate failure loads of the TCL and TMMA techniques were significantly greater than that of the TSS technique but not significantly greater than that of the TSK technique. However, the TSK technique causes minimal iatrogenic injury to the fragile meniscal root because it only requires one piercing per loop, whereas the TMMA technique requires multiple piercings, increasing meniscus morbidity and the risk of suture cutout.

The TSS technique is widely accepted as the standard for meniscal root repair due to its simplicity and strength. However, its lower yield load and ultimate failure load may not be optimal for those requiring higher load‐bearing capacity or greater stability in meniscus root repair. The findings of this study are consistent with those of biomechanical studies that indicate that less‐invasive suturing methods involving minimal penetration of meniscus tissue may offer superior resistance against displacements [2, 4, 8, 21]. Most surgeons prefer a suture construct that is technically less demanding but provides high tensile strength and minimal displacement. The TSK technique represents a viable alternative, increasing suture strength without increasing structural complexity or compromising displacement resistance.

There were some differences between the results of this study and those reported in previous studies. Specifically, for the TSS technique, the difference between the pre‐ and postconditioning stiffness values in the present study (25.25 N/mm) fell between the values (between 20.6 and 31.5 N/mm) reported by Anz et al. [1] but was similar to the value reported by Vertullo et al. (24.5 N/mm) [1, 34]. Emulating the biomechanical testing protocol of Vertullo et al. [34], the present study also used separate programs for cyclic loading and load‐to‐failure tests to create a temporal gap between assessments for measured construct relaxation. Finally, the yield and ultimate failure loads for the TSS technique in the present study were lower than those reported by Anz et al., Feucht et al., LaPrade et al., and Okimura et al. [1, 8, 21, 27]. This discrepancy may be attributable to the use of older cadavers in the present study.

## LIMITATION

As with other in vitro biomechanical investigations, this study has several inherent limitations. First, the results only accounted for stability at time zero; they could not incorporate the gradual healing effect that likely occurs during the initial 6–8 weeks of nonweight‐bearing postoperative rehabilitation [3]. Second, the forces applied during testing were uniaxial only and did not replicate the vertical shear, radial extrusion, or axial compression forces that a meniscal root would sustain under natural knee motion and loading. Although an in vivo study would better emulate the physiologic force vectors to the meniscal root, the objective of this study was merely to compare the strength of different meniscal repair constructs in a simple and reproducible fashion. Third, the average age of the cadaveric specimens exceeded the typical age range of individuals undergoing meniscal repair in clinical settings [6, 33, 35], primarily because specimens from younger individuals are seldom accessible. Last, the use of both medial and lateral meniscal tissue in each group might present a confounding bias to the results; however, Kopf et al. [15] reported that the difference between the native strength of the posterior medial and posterior lateral roots was not significant.

## CONCLUSION

The slip‐knot technique has proven to be a simple, yet robust and stable meniscal root fixation option; moreover, it exhibited superiority over the more complex modified Mason–Allen suture construct in resisting displacement at the ultimate failure load.

## AUTHOR CONTRIBUTIONS

**Ting‐Yu Chang**: Formal analysis and investigation; writing—original draft preparation. **Tai‐Hua Yang**: Methodology; cadaver resources and study supervision. **Kuan‐Yu Lin**: Conceptualization; writing—review and editing.

## CONFLICT OF INTEREST STATEMENT

The authors declare no conflict of interest.

## ETHICS STATEMENT

This study utilized human tissue (menisci) that were procured via the biobank of institution (National Cheng Kung University, Tainan, Taiwan), which provides de‐identified samples. This study was reviewed and deemed exempt by the Institutional Review Board. The biobank protocols are in accordance with the ethical standards of our institution and with the 1964 Helsinki declaration and its later amendments or comparable ethical standards.

## Data Availability

The data that support the findings of this study are available on request from the corresponding author. The data are not publicly available due to privacy or ethical restrictions.
